# Effects of small-dose S-ketamine on anesthesia-induced atelectasis in patients undergoing general anesthesia accessed by lung ultrasound: study protocol for a randomized, double-blinded controlled trial

**DOI:** 10.1186/s13063-023-07779-y

**Published:** 2024-01-18

**Authors:** Di Zhang, Yi Liang, Di Bao, Wei Xiong, Lu Li, Yaxin Wang, Bin Liu, Xu Jin

**Affiliations:** Department of Anesthesiology, Beijing Tiantan Hospital, Capital Medical University, Beijing, 100070 China

**Keywords:** Atelectasis, S-ketamine, Ultrasound, Randomized controlled trial, Protocol

## Abstract

**Background:**

Atelectasis after anesthesia induction in most patients undergoing general anesthesia may lead to postoperative pulmonary complications (PPCs) and affect postoperative outcomes. However, there is still no existing effective method used for the prevention of perioperative atelectasis. S-ketamine may prevent atelectasis due to airway smooth muscle relaxation and anti-inflammatory effects. Lung ultrasound is a portable and reliable bedside imaging technology for diagnosing anesthesia-induced atelectasis. The primary objective of this study is to assess whether a small dose of S-ketamine can reduce the incidence of atelectasis after intubation, and further investigate the effects of preventing the early formation of perioperative atelectasis and PPCs.

**Methods:**

This is a single-institution, prospective, randomized controlled, parallel grouping, and double-blind study. From October 2020 to March 2022, 100 patients (18–60 years old) scheduled for elective surgery will be recruited from Beijing Tiantan Hospital, Capital Medical University, and randomly assigned to the S-ketamine group (group 1) and the normal saline group (group 2) at a ratio of 1:1. The label-masked agents will be administered 5 min before induction, and all patients will undergo a standardized general anesthesia protocol. Related data will be collected at three time points: after radial artery puncture (T1), 15 min after tracheal intubation (T2), and before extubation (T3). The primary outcome will be the total lung ultrasound scores (LUS) at T2. Secondary outcomes will include LUS in six chest regions at T2, total LUS at T3, arterial blood gas analysis results (PaCO_2_, PaO_2_) and PaO_2_/FiO_2_ at T2 and T3, and plateau pressure (P_plat_) and dynamic lung compliance (Cdyn) at T2 and T3. The incidence of postoperative complications associated with S-ketamine and PPCs at 2 h and 24 h after surgery will be recorded.

**Discussion:**

This trial aims to explore whether a simple and feasible application of S-ketamine before the induction of general anesthesia can prevent atelectasis. The results of this study may provide new ideas and direct clinical evidence for the prevention and treatment of perioperative pulmonary complications during anesthesia.

**Trial registration:**

ClinicalTrials.gov NCT04745286. Registered on February 9, 2021.

**Supplementary Information:**

The online version contains supplementary material available at 10.1186/s13063-023-07779-y.

## Background

Atelectasis refers to the complete or partial collapse of the entire lung or an area of the lung. Previous studies have reported that the incidence of pulmonary atelectasis during general anesthesia is as high as 85–90% [1]. It usually appears a few minutes after the application of 100% oxygen for pre-oxygenation and induction and continues until after surgery [1, 2]. Atelectasis plays an important role in the intrapulmonary shunt increase [3], gas exchange abnormalities, hypoxemia, and compliance decrease. These are associated with postoperative pulmonary complications (PPCs). It seriously affects the postoperative outcome in surgical patients [4–7].

The mechanism of perioperative atelectasis remains unclear. At present, the following three mechanisms have been mainly considered: compressive atelectasis, gas absorption, and alveolar surfactant destruction [2, 8–13]. The mechanism of atelectasis after anesthesia induction is mainly considered to be related to small airway closure and gas absorption in pre-oxygenated alveoli [14].

Ketamine and its S-isomer have been widely used in clinical anesthesia, especially in patients with sepsis or cardiovascular instability [15]. Ketamine and its S-isomer achieve anesthesia and analgesia primarily through non-competitive antagonism at N-methyl-d-aspartate receptors. For the different effects of other anesthetics, S-ketamine has effects on relaxing the bronchial smooth muscles and inhibiting inflammation, which may prevent atelectasis after anesthesia induction. Studies have shown that ketamine can relax bronchial smooth muscles in order to alleviate the closure of small airways, thereby significantly reducing ventilation resistance [16–18]. This effect is especially beneficial for patients with asthma and bronchospasm. Ketamine has a unique pleiotropic anti-inflammatory effect [19–22], which can prevent exacerbated pro-inflammatory reactions and extension of local inflammation mainly by inducing anti-inflammatory mediators such as adenosine [23]. These anti-inflammatory mediators inhibit the activation of nuclear factor NF-κB and repress the release of pro-inflammatory cytokines such as TNF-α, IL-6, and IL-8 [18, 23, 24]. In addition, it suppresses the activation and recruitment of leukocytes and precipitates the apoptosis of inflammatory cells in the lungs [25–27].

Among all the detection methods of pulmonary disease, lung ultrasound, as a bedside, non-invasive, diagnostic tool, has been increasingly used in the assessment of atelectasis during surgery. Furthermore, it has advantages such as convenience and rapidity and it is radiation-free [28–31]. It has a reliable sensitivity of 93% and specificity of 100% [32].

Previous studies on atelectasis during anesthesia mostly focused on the influence of respiratory parameters such as tidal volume, respiratory rate, inhaled oxygen concentration, positive end-expiratory pressure (PEEP), and the effects of manipulation or mechanical expansion on postoperative atelectasis [7, 33–35]. To date, there is only a little evidence available for pre-treatment with medicine for atelectasis during general anesthesia. The primary objective of this study is to assess whether a small dose of S-ketamine can reduce the incidence of atelectasis after intubation, and further investigate the effects of preventing the early formation of perioperative atelectasis and PPCs.

## Methods

### Trial design and study setting

**Fig. 1:**
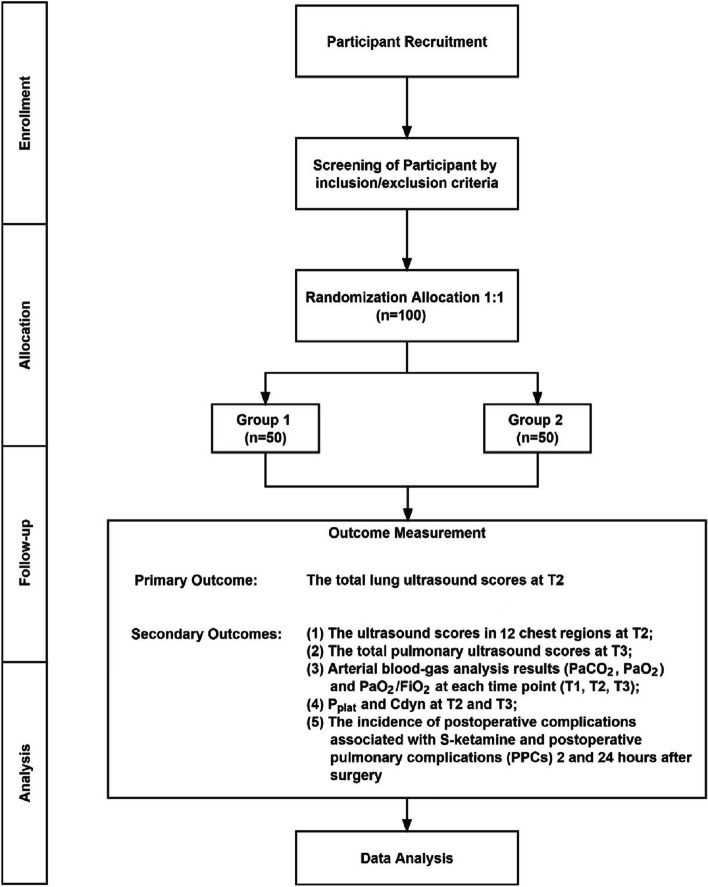
Flow diagram of the Consolidated Standards of Reporting Trials [36]. Group 1, S-ketamine group; group 2, normal saline group; T1, after radial artery puncture; T2, 15 min after tracheal intubation; T3, before extubation

This is a single-center, prospective, double-blind, parallel grouping, randomized controlled trial conducted at Beijing Tiantan Hospital, Capital Medical University, from October 2020 to December 2022. This study was approved by the Institutional Review Board of the Capital Medical University Hospital Institutional Review Board (IRB) and registered at ClinicalTrials.gov (trial registration number: NCT04745286). The patient flow diagram of the study is presented in Fig. 1, and the trial schedule is shown in Table 1.

**Table 1:** The schedule of enrollment, interventions, and assessments

	**Study period**						
	Enrollment	Allocation	Postallocation				
Time point	Day − 1	Day 0	T1 (after radial artery puncture)	15 min after intubation (T2)	Before extubation (T3)	2 h after surgery	24 h after surgery
Enrollment							
Eligibility screen	X						
Informed consent	X						
Randomization		X					
Allocation		X					
Interventions							
[Group 1] S-ketamine group			X				
[Group 2] Normal saline group			X				
Outcome assessment							
Total LUS			X	※	X		
LUS of 12 chest regions				X			
PaCO_2_, PaO_2_, and PaO_2_/FiO_2_			X	X	X		
P_plat_ and Cdyn				X	X		
Incidence of postoperative complications associated S-ketamine						X	X
Incidence of PPCs						X	X

*LUS* lung ultrasound scores, *P*_*plat*_ plateau pressure, *Cdyn* dynamic lung compliance, *PPCs* postoperative pulmonary complications. ※ primary outcome

### Eligibility

The trial investigator will identify consecutive eligible patients according to the inclusion and exclusion criteria on the day before the operation. When the patients meet the eligibility criteria, the investigators will explain the study plan and relevant information to the participants.

#### Inclusion criteria

1. Age of 18–60 years
2. American Society of Anesthesiologists (ASA) physical status of I–II
3. Patients scheduled for urinary, obstetrics, and gynecology surgery in the supine or lithotomy position under general anesthesia with expected operation time of more than 1 h
4. Patients and their authorized surrogates will sign an informed consent form

#### Exclusion criteria

1. Patients in whom the application of lung ultrasound is difficult, such as patients with chest fractures or a history of surgical
2. Patients with a history of upper respiratory tract infection 1 month before surgery or a history of smoking more than 6 packs/year
3. When the preoperative chest X-ray or CT results are abnormal, including atelectasis, pneumothorax, pleural effusion, or pneumonia
4. BMI > 30kg/m.^2^
5. Expected difficulties in intubation or mask ventilation
6. Patients with a significant increase in intraocular pressure and intracranial pressure before surgery
7. Patients who are allergic to S-ketamine, propofol, and opioids
8. Patients with a history of psychiatric disorders

#### Dropout criteria

1. Unexpected retention of the tracheal tube after operation
2. Receiving a second operation during hospitalization
3. Perioperative complications such as pneumothorax and subcutaneous emphysema which make lung ultrasound assessment difficult
4. Withdrawal during the trial

### Allocation, randomization, and blinding

The study investigator will be trained to implement standardized intervention methods.

#### Sequence generation

An independent statistician will perform randomization using a computer random number list generated by Stata software version 15.1. Participants will be randomly assigned to either the S-ketamine group (group 1) or the normal saline group (group 2), and the allocation ratio will be 1:1.

#### Allocation concealment mechanism

Group allocations will be sealed in sequentially numbered opaque envelopes. An independent study researcher who is not involved in anesthesia management or data collection will obtain randomized allocation information and prepare the study agents in the opaque 5-mL syringes before surgery. The blinded anesthesiologists in charge will use the study agents during anesthesia induction to complete the intervention and record the intraoperative data. Two well-trained anesthesiologists will perform lung ultrasound assessments and record LUS at relevant time points. Blinded and trained research assistants will perform the postoperative follow-up 24 h after the operation to record relevant data. All deviations or missing data will also be recorded.

#### Blinding

In this study, anesthesiologists in charge, patients, lung ultrasound assessors, and follow-up staff will be blinded to the randomized information. After all the results are completely analyzed, the blinding will be stopped.

### Criteria for discontinuing or modifying the allocated interventions

The assignment will not be modified; however, the study will be terminated in the following situations: (1) intraoperative serious adverse events, such as hemorrhage, shock, and malignant hyperthermia, and (2) without anticipated difficult airway or failure to extubation after surgery.

### Data collection and management

All the study-related data of recruited patients will be recorded in an electronic storage system. These data will include baseline information, intraoperative and postoperative follow-up information, and lung ultrasound-related data. The data of each patient will be entered into the EpiData database using a dual data entry system. The data will not be disclosed to other researchers until the study is completed. An inspector will regularly check and revise these records to ensure data quality.

### Interventions

Patients will be intravenously infused with the label-covered trial agent 5 min before induction, and all patients will undergo a standardized general anesthesia and analgesia protocol. Lung ultrasound scans will be performed at three points in each patient: immediately after radial artery puncture (T1), 15 min after tracheal intubation (T2), and before extubation (T3). Three arterial blood samples will be obtained through radial artery puncture at each time point (T1, T2, T3), and blood gas measurements will be performed immediately using a standard technique (ABL800).

Agent preparation method: S-ketamine group (group 1): S-ketamine (50 mg; 2 mL) will be diluted with normal saline to 5 mL in an opaque 5-mL syringe (10 mg mL^−1^). Normal saline group (group 2): 5 mL of normal saline. Both will be labeled as “study agents” and injected with a dose of 0.025 mL kg^−1^.

### Anesthesia and analgesia

Standard monitoring of non-invasive blood pressure (BP), heart rate (HR), and pulse oxygen saturation (SpO_2_) will be commenced upon the patient’s arrival at the operating room. Radial artery puncture will be performed under local anesthesia to perform blood gas sampling and invasive arterial blood pressure (ABP) monitoring. Patients will receive midazolam 2 mg intravenously as a premedication 5 min before induction. Anesthesia will be induced by pre-oxygenation with 100% oxygen inhalation for 3 min via a face mask. This will be followed by an intravenous administration of sufentanil 0.3–0.5 μg kg^−1^, propofol 1.5–2.5 mg kg^−1^, and rocuronium 0.6–1.0 mg kg^−1^. After spontaneous breathing disappears, the volume control mode will be used to control the patient’s breathing through the mask for 2 min, with a tidal volume of 6–8 mL kg^−1^ (predicted body weight without PEEP), ventilatory frequency of 12 bpm, and 100% oxygen concentration. It takes approximately 5–7 min from pre-oxygenation to start intubation, and then we intubate with a suitably sized tracheal tube with a cuff. All patients will receive mechanical ventilation with a FiO_2_ of 0.4 and an inspiratory-to-expiratory ratio of 1:1.5–2. The ventilatory parameters will be adjusted to maintain the end-tidal carbon dioxide partial pressure (P_ET_CO_2_) within 35–40 mmHg and airway pressure not exceeding 25 cm H_2_O.

Anesthesia will be maintained with continuous intravenous infusion of remifentanil 0.1–0.2 μg kg^−1^ min^−1^ and propofol 3–6 mg kg^−1^ h^−1^, combined with inhalation of 0.5–2 MAC sevoflurane. Sufentanil 0.05–0.1 µg kg^−1^ will be administered intermittently in accordance with the demand for analgesia. The mean arterial pressure and HR will be maintained within 30% of the baseline value. The patient’s axillary temperature will be maintained at 36–37 °C throughout the operation. During anesthesia, ABP, P_ET_CO_2_, peak inspiratory pressure (PIP), and plateau pressure (P_plat_) will be monitored. All anesthetics will be discontinued at the end of the surgery.

### Pulmonary ultrasonography

A well-trained blinded anesthesiologist will perform all lung ultrasound scans using SonoSite M-Turbo with a 5–12-MHz linear transducer. Patients will be scanned in the supine position following the lung ultrasound method described by Sun et al. [37]. Briefly, six regions will be scanned in each hemithorax. Scans 1 and 2 will be performed in the clavicle midline, scans 3 and 4 will be performed in the axillary midline, and the intercostal scans (scans 5 and 6) will be performed at the fifth and sixth intercostal spaces in the posterior axillary line. A linear probe will be placed parallel to the ribs, and the intercostal space of each region will be scanned sequentially from right to left, cranial to caudal, and anterior to posterior within 2 min.

The video clips of each region will be stored on a portable hard drive for offline analysis. Scoring criteria of B-lines are as follows: B-lines will be categorized into four grades (0–3): B0, normally ventilated areas with lung sliding sign: A-line and isolated B-lines (< 3); B1, mild reduction in lung ventilation/moderate loss of lung tissue gasification: B-lines (≥ 3) with clear boundary, regular distribution, and spacing ≥ 7 mm, or irregular, clear interval; B2, severe reduction in lung ventilation/severe lung gasification: multiple coalescent B-lines spaced ≤ 3 mm and continuous fusion; and B3, atelectasis/pulmonary consolidation: tissue-like signs, fragment signs, and bronchial inflation signs appear.

### Outcome measurement

#### Primary outcome

Total LUS at T2.

#### Secondary outcomes

LUS in 12 chest regions (scans 1-6 in each hemihorax) at T2; total LUS at T3; arterial blood gas analysis results (PaCO_2_, PaO_2_) and PaO_2_/FiO_2_ at T2 and T3, P_plat_ and dynamic lung compliance (Cdyn) [Cdyn = tidal volume/(PIP-PEEP)] at T2 and T3; the incidence of postoperative complications associated with S-ketamine at 2 h and 24 h after surgery (visual impairment, dizziness, pathological irritability, nightmares, and hallucinations); the incidence of PPCs at 2h and 24 h after surgery (the clinical outcome definitions of PPCs will adopt European joint taskforce guidelines published in 2015 [38]. These include respiratory infection, respiratory failure, atelectasis, pleural effusion, pneumothorax, bronchospasm, aspiration pneumonia, pulmonary edema, ARDS, tracheobronchitis, pulmonary edema, exacerbation of pre-existing lung disease, pulmonary embolism).

#### Other indicators to be recorded

1. Baseline value

General information of patients: age, height, weight, BMI, smoking history, ASA, type of operation, complications, types, and severity of complications.

Anesthesia-related variables: duration of apnea during artificial airway establishment (recorded by stopwatch), laryngoscope insertion times, liquid volume (from entering the room to T2).

Intraoperative indicators: total LUS at T1; arterial blood gas analysis results (PaCO_2_, PaO_2_) and PaO_2_/FiO_2_ at T1; mechanical ventilation parameters (tidal volume, ventilation frequency, and FiO_2_).

(2) Other indicators

Vital signs (pulse, BP, HR, and SpO_2_) at each time point (T1, T2, and T3).

### Dissemination

The study protocol was registered at ClinicalTrials.gov (identified as NCT04745286). The results will be available and disseminated to all participants, investigators, and healthcare providers in the form of summary documents, presentations on the Internet. The data set analyzed in the current study will be obtained from the corresponding author upon reasonable request.

### Sample size calculation and statistical analysis

According to previous studies, the incidence of atelectasis is approximately 90% in patients pre-oxygenated with 100% oxygen within a few minutes after anesthesia [1, 4, 33, 39]. We estimate that the incidence of atelectasis is 85%, and the preoperative administration of S-ketamine can reduce the incidence of atelectasis to 60%. The sample size was calculated by PASS 15.0. Finally, 50 cases in each group will be estimated to provide 80% power with a significance level *α* of 0.05, allowing for a dropout rate of 5%.

Statistical analysis will be performed using SPSS 24.0. Values will be expressed as mean ± standard deviation ($\overline{x }$±s), median and interquartile range (IQR), and percentages according to different distribution types. After testing for normality of continuous variables, Student’s *t*-test or the Mann–Whitney *U*-test will be used for comparison between and within groups, as appropriate. Categorical variables will be analyzed using the chi-square test or Fisher’s exact test. All statistical analyses in our study will be conducted with a bilateral test, and statistical significance will be set at *P* < 0.05. We will exclude patients whose primary data are missing. Both intention-to-treat analysis and per-protocol analysis will be conducted in this study.

## Discussion

In this prospective randomized controlled trial, we intend to verify whether using a small-dose S-ketamine before induction can alleviate atelectasis 15 min after tracheal intubation under general anesthesia and to further study the effect of low-dose S-ketamine on PPCs. To our knowledge, to date, this is the only randomized clinical trial of preoperative agents on atelectasis. This will possibly provide a simple and feasible new method on agent prevention and direct clinical evidence for anesthesiologists to conduct intraoperative lung protection strategies. We will use the new non-invasive lung ultrasound technology as a guide for the diagnosis of atelectasis, which has many advantages, such as not being restricted by time or venue and without radiation. The reasons for choosing lung ultrasound scores (LUS) at 15 min after endotracheal intubation as the primary outcome are as follows: (1) previous studies showed that atelectasis has already occurred at this moment after induction and pre-oxygenation with 100% oxygen [40]; (2) this period is within the active duration of a single small dose of S-ketamine, and the effect of operation time and extubation on atelectasis can be excluded; (3) lung ultrasound can detect atelectasis in real-time, and LUS can accurately reflect the pulmonary state. In addition, to analyze the effects of the intervention on gas exchange (PaCO_2_, PaO_2_, and PaO_2_/FiO_2_) in detail, we performed blood gas measurements at three time points (T1, T2, T3). There are limitations in this trial: (1) we will study only the effects of a small dose of ketamine on anesthesia-induced atelectasis, but will not compare the effects of different concentrations of ketamine; (2) ultrasound assessment depends on the operator and reviewer, which may cause subjective bias. However, in order to reduce this bias, all the researchers involved in the pulmonary assessment will be blinded to the allocated information, and all intercostal spaces will be carefully scanned to avoid missing any abnormal findings. In addition, the ultrasound images for each lung region will be analyzed by two experienced radiologists; (3) this is a single-center trial and may have selection bias.

### Supplementary Information

**Additional file 1.**

## Data Availability

The CRF will be completed by a blinded researcher, and the ultrasound equipment will be calibrated uniformly. Every 20 cases completed in the experiment will be checked by the Data Monitoring Committee and stored in the security database of the Department of Anesthesiology, Beijing Tiantan Hospital, Capital Medical University. The study protocol is available on ClinicalTrials.gov ( identifier: NCT04745286), and the research results, analyzed data sets, etc., can be made available upon reasonable request from the corresponding author.

## Abbreviations

PPCs: Postoperative pulmonary complications
V_A_/Q: Ventilation/perfusion
PEEP: Positive end-expiratory pressure
LUS: Lung ultrasound scores
IRB: Institutional Review Board
ASA: American Society of Anesthesiologists
BP: Blood pressure
HR: Heart rate
SpO_2_: Pulse oxygen saturation
ABP: Arterial blood pressure
P_ET_CO_2_: End-tidal carbon dioxide partial pressure
PIP: Peak inspiratory pressure
P_plat_: Plateau pressure
Cdyn: Dynamic lung compliance
CRF: Case report form
PI: Principal investigator
